# M2 macrophages predict worse long-term outcomes in human acute tubular necrosis

**DOI:** 10.1038/s41598-020-58725-w

**Published:** 2020-02-07

**Authors:** Myung-Gyu Kim, Kijoon Lim, Yoo Jin Lee, Jihyun Yang, Se Won Oh, Won Yong Cho, Sang-Kyung Jo

**Affiliations:** 1 Division of Nephrology, Department of Internal Medicine, Korea University Anam Hospital, Seoul, Republic of Korea; 2 Department of Pathology, Korea University Anam Hospital, Seoul, Republic of Korea

**Keywords:** Prognostic markers, Acute kidney injury

## Abstract

Although macrophages are important players in the injury/repair processes in animal models of acute kidney injury (AKI), their roles in human AKI remains uncertain owing to a paucity of human biopsy studies. We investigated the role of macrophages in 72 cases of biopsy-proven acute tubular necrosis (ATN) and six cases of healthy kidney. Macrophages were identified by CD68 and CD163 immunohistochemistry and analyzed for their effect on renal outcomes. CD163+ M2 macrophages outnumbered CD68+ cells in the healthy kidneys, suggesting that CD163+ macrophages are resident macrophages. The infiltration of both subtypes of macrophages increased significantly in ATN. The density of the CD68+ macrophages was significantly higher in advanced-stage AKI, whereas CD163+ M2 macrophages was not. Eighty percent of patients exhibited renal functional recovery during follow-up. Older age and a higher density of CD163+ macrophages predicted non-recovery, whereas the AKI stage, tubular injury score, and density of CD68+ cells did not. The density of CD163+ M2 macrophages was an independent predictor of low eGFR at 3 months in advanced-stage AKI. This is the first human study demonstrating the possible role of macrophages in the injury and repair phases of AKI.

## Introduction

Acute kidney injury (AKI) is an important health burden with high morbidity and mortality rates, especially in the elderly or those with chronic illnesses^1,2^. The cellular and molecular mechanisms underlying AKI have been identified through numerous experimental studies, and efforts to develop novel therapeutics are still ongoing. However, lack of biopsies in human AKI has created a huge knowledge gap, and therefore has delayed the effective translation of animal studies into human AKI^3^.

In contrast, translational studies in cancer research using human tissues have led to the development of multiple cancer therapies based on genetic information, and personalized therapies have recently become popular^4^. Therefore, a better understanding of human AKI using biopsy samples is urgently needed in order to develop novel preventive and therapeutic strategies.

Inflammation is an important player in animal models of AKI^5^. Neutrophils, natural killer T cells, monocyte/macrophage/dendritic cells, and T cells are involved in the initial inflammatory process of AKI; notably, macrophages play a complex role in both injury and subsequent recovery processes^6,7^. Recently, it has been shown that the conversion of pro-inflammatory (M1) to anti-inflammatory (M2) macrophage phenotypes promotes renal repair, and an imbalance between M1 and M2 polarization can lead to renal fibrosis^8,9^. However, it should be noted that most of this work has been carried out using animal models of AKI, and only a few studies have been conducted on human AKI.

In the present study, we reviewed the records of 72 human acute tubular necrosis (ATN) biopsy samples to investigate the possible roles of different macrophage subsets in injury and recovery during AKI. We found that a higher CD163+ M2 macrophage density was associated with poor long-term renal outcome.

## Results

### Patients’ characteristics

Table 1 shows the patients’ baseline characteristics. The mean age was 51.9 ± 14.2 years, and 72% were male. The peak serum creatinine level was 4.61 ± 4.34 mg/dL, and the average time from peak creatinine to biopsy was 3.27 ± 0.49 days. Of the patients, 39 (54%) patients had stage 3 AKI, and 28.4% required temporary dialysis. A significantly higher percentage of patients with native kidney ATN had stage 3 AKI (86.2 vs 16.3% native kidney vs deceased donor kidney, *p* < 0.01) with significantly higher peak serum creatinine (7.9 ± 5.2 vs 2.4 ± 1.3 mg/dL, *p* < 0.01) (Table 1). The most common etiologies leading to ATN in the native kidneys were ischemia (24.1%), followed by multifactorial etiologies (20.7%), infection (17.2%), and nephrotoxicants (13.8%).

**Table 1:** Basal characteristics of the patients.

	Total (n = 72)	Native AKI (n = 29)	Deceased donor AKI (n = 43)	P-value
Age (years)	51.89 ± 14.18	48.28 ± 15.36	54.33 ± 12.95	0.076
Sex (M, %)	72.2	72.4	72.1	0.976
DM (%)	29.2	20.7	34.8	0.194
HTN (%)	33.3	34.5	32.5	0.865
Baseline Cr (mg/dL)	1.10 ± 0.48	0.94 ± 0.18	1.21 ± 0.59	0.662
Peak Cr to Bx (day)	3.27 ± 4.17	5.03 ± 4.56	2.09 ± 3.46	0.003
Peak Cr (mg/dL)	4.61 ± 4.34	7.89 ± 5.15	2.39 ± 1.33	<0.001
Stage 3 AKI (n, %)	32 (44.4)	25 (86.2)	7 (16.3)	<0.001
Dialysis (n, %)	21 (29.2)	13 (44.8)	8 (18.6)	0.020
Mortality (n, %)	8 (11.1)	3 (10.3)	5 (11.6)	0.865
f/u duration (days)	1063.05 ± 928.20	1354.27 ± 1346.23	866.65 ± 387.54	0.028
AKI recovery (%)	80.5	82.7	79.0	0.698

Values are presented as n (%) or mean ± standard deviation.

Abbreviations: DM, diabetes mellitus; HTN, hypertension; Bx, biopsy; AKI, acute kidney injury; Cr, creatinine; f/u, follow-up.

### Macrophages in healthy kidneys

**Figure 1:**
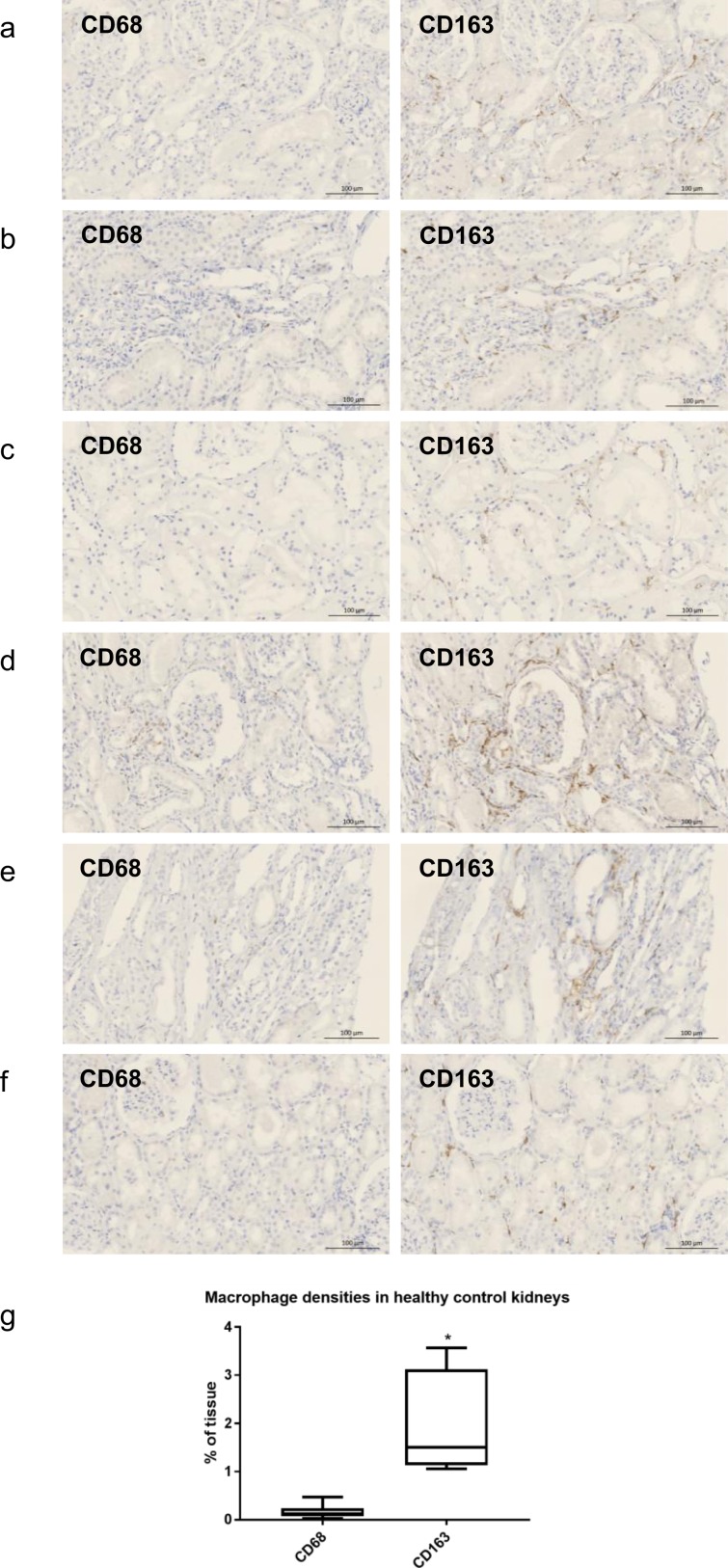
(**a**–**f**) Representative images of immunostaining with anti-CD68 and anti-CD163 in six control patients with normal histology. CD163+ cells were found in the interstitium of the normal kidneys, but CD68+ cells were very rare. (**g**) The infiltration density of the CD163+ cells was significantly higher than that of the CD68+ cells. Magnification: x100 (bar = 100 μm), *p < 0.05 compared to CD68+ cells by two-tailed t-test.

The renal tissues of six patients with microscopic hematuria but normal histology were stained with anti-CD68 and anti-CD163. In the healthy kidneys, CD163+ macrophages showed a spindle-shaped morphology and were interspersed in the interstitial area (1.96 ± 0.43% area), whereas only very few CD68+ cells were found (0.17 ± 0.06% area), suggesting that CD163+ cells represent the resident macrophages in the kidney (Fig. 1).

### Macrophages and severity of AKI

**Figure 2:**
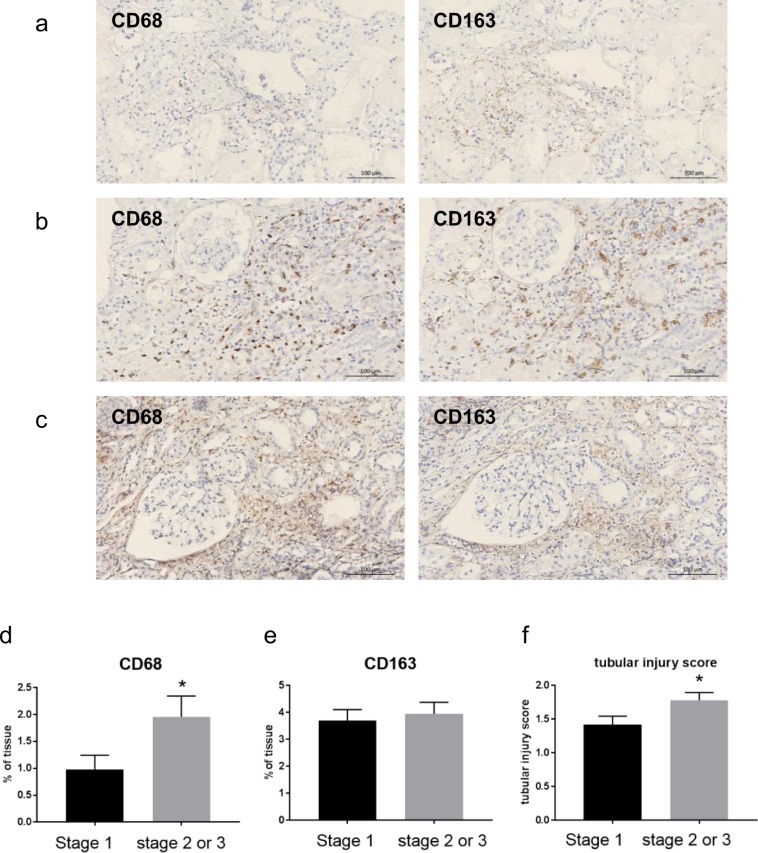
Representative images of immunostaining with anti-CD68 and anti-CD163 in ATN. Compared to healthy control kidneys, both CD68+ and CD163+ cell densities increased. (**a**) Stage 1 AKI. (**b**) Stage 2 AKI. (**c**) Stage 3 AKI. (**d**) Density of CD68+ cells according to stage of AKI. (**e**) Density of CD163+ cells according to stage of AKI. (**f**) Tubular-injury score according to stage of AKI. Magnification: x100 (bar = 100 μm), *p < 0.05 compared to stage 1 by two-tailed t-test.

The densities of both CD68+ and CD163+ macrophages increased significantly in ATN (CD68, 0.17 ± 0.06 vs 1.60 ± 2.29% area, *p* < 0.01; CD163, 1.96 ± 0.43 vs 3.86 ± 2.60% area, *p* < 0.01) (Fig. 2). Because CD68+ cells are rarely observed in healthy kidneys, these cells in ATN are likely to be monocytes that have migrated from the circulation after injury.

Because CD68 is expressed on some non-myeloid cells, such as fibroblasts/stromal cells^8,9^, we also carried out α-smooth-muscle actin (α-SMA) immunohistochemistry. α-SMA immunoreactivity was mostly found in glomeruli and arterioles, as well as in some tubular basement membrane, but not in the interstitium where CD68+ cells were found. These results suggest that interstitial CD68+ cells are likely to be macrophages (Supplementary Fig. S1).

When we compared the density of CD68 or CD163 cells according to the severity of AKI, we observed that the density of CD68+ cells in stage 2 or 3 severe AKI was significantly higher than that in stage 1. However, the density of CD163+ cells did not differ according to the stage of AKI (Fig. 2a–e). The tubular-injury score in stage 2 or 3 AKI was also significantly higher than that in stage 1 AKI (Fig. 2f).

### Macrophages and recovery from AKI

**Figure 3:**
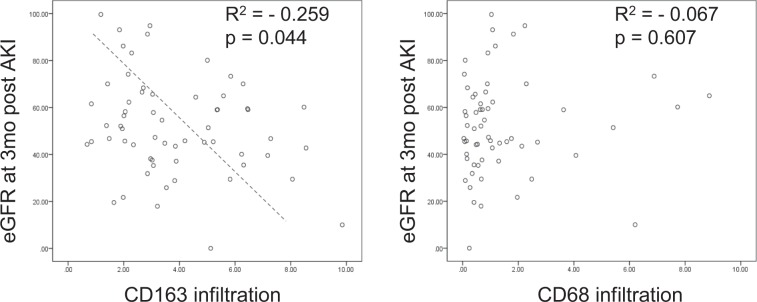
Linear correlation between CD163+ or CD68+ cell infiltration and 3-month eGFR in stage 2 or 3 acute kidney injury (AKI).

During the mean follow-up period of 35.4 ± 30.9 months, 80.5% of the patients achieved renal functional recovery, defined as recovery to within 25% of baseline estimated glomerular filtration rates (eGFR). Stage of AKI, tubular-injury score, or density of CD68+ macrophages did not predict recovery. However, a higher density of CD163+ macrophages was associated with non-recovery (Table 2). In the subgroup analysis, this association was evident in stage 2 and 3 advanced AKI, and also in AKI with biopsy time from peak creatinine >2 days (Supplementary Tables S1,S2), suggesting that persistent infiltration of M2 macrophages may have a negative effect on recovery. Finally, there was a negative correlation between the density of CD163+ macrophages at the time of biopsy and the eGFR at 3 months. In particular, multivariate analysis showed an independent correlation between them in patients with stage 2 or 3 AKI, regardless of the patient’s gender, the presence of diabetes or hypertension, or the stage of AKI (Fig. 3, Tables 3 and 4).

**Table 2:** Factors affecting renal outcomes.

	1-month eGFR			3-month eGFR			AKI recovery*		
	eGFR <45	eGFR >45	P-value	eGFR <45	eGFR >45	P-value	Non-recovery	Recovery	P-value
Stage 2 or 3 (%)	69.6	60.4	0.600	54.5	59.0	0.792	64.3	63.8	0.973
Tubular injury score	1.83 ± 0.78	1.56 ± 0.74	0.172	1.73 ± 0.70	1.49 ± 0.72	0.212	1.93 ± 0.73	1.59 ± 0.75	0.128
CD68 (% area)	1.95 ± 2.36	1.42 ± 2.29	0.368	1.19 ± 1.48	1.56 ± 2.13	0.476	1.72 ± 2.17	1.58 ± 2.34	0.831
CD163 (% area)	4.89 ± 2.85	3.22 ± 2.10	0.007	4.43 ± 2.40	3.52 ± 1.98	0.116	5.61 ± 3.24	3.44 ± 2.27	0.004

Values are presented as n (%) or mean ± standard deviation.

Abbreviations: eGFR, estimated glomerular filtration rates; AKI, acute kidney injury.

*AKI recovery was defined as recovery of renal function to within 25% of baseline eGFR during follow-up.

**Table 3:** Univariate analysis of risk factors for predicting 3-month eGFR post AKI.

Risk factors	Total		Stage 2 or 3	
	β	P-value	β	P-value
Age	−0.423	0.001	−0.379	0.025
DM	−0.242	0.061	−0.237	0.171
HTN	−0.056	0.669	0.069	0.695
AKI stage	0.066	0.613	0.005	0.977
CD163	−0.259	0.044	−0.401	0.017
CD68	0.067	0.607	−0.011	0.950
Tubular injury score	−0.175	0.561	−0.129	0.461

Simple linear regression analysis between risk factors and 3-month eGFR.

Abbreviations: DM, diabetes mellitus; HTN, hypertension; AKI, acute kidney injury.

**Table 4:** Multivariate analysis of risk factors for predicting 3-month eGFR post AKI in stage 2 or 3 AKI.

	β	P-value	95% CI
CD163	−4.252	0.017	−7.692-0.812

In the multivariate analysis model, a backward (LR) selection approach was adopted. Age, diabetes mellitus (DM), hypertension (HTN), and severity of acute kidney injury (AKI) are not displayed.

Abbreviations: CI, Confidence interval.

## Discussion

Our data demonstrated the increase of both CD68+ and CD163+ macrophages in human ATN. There was a positive correlation between the density of CD68+ macrophages and the severity of AKI, whereas the density of CD163+ M2 macrophages was associated with a lack of renal functional recovery. To the best of our knowledge, this study is the first demonstration of the important role of macrophages in human AKI.

Macrophages are heterogeneous mononuclear phagocytes with substantial plasticity. In animal models of IRI, infiltrating monocytes/macrophages, and the activation of resident macrophages/dendritic cells contribute to early inflammation and injury^10^. However, they also participate in the repair and healing process via a phenotypic shift from classically activated M1 to alternatively activated M2 macrophages^11^. There is also evidence that an imbalance between M1 and M2 macrophages may lead to tubulointerstitial fibrosis^12,13^. The dynamics and distinct roles of macrophages have been clarified in animal models, but the role of macrophages in human AKI remains uncertain, owing to a paucity of kidney biopsy-based studies.

In our study of 72 cases of biopsy-proven ATN, we identified two different subsets of macrophages in the kidneys—i.e., CD68+ and CD163+ cells—and demonstrated their possible role in human AKI. In selecting native-kidney ATN cases, we excluded those with tubular atrophy or interstitial fibrosis because of the possible confounding effects of macrophages on chronic lesions. Deceased donor ATN cases that subsequently developed biopsy-proven rejection during the initial 6 months were also excluded, because rejection might have affected the recovery process following ATN.

We first examined the macrophages in the healthy control kidneys. In contrast to the CD68+ macrophages, which were present in very low numbers, the CD163+ macrophages were more prevalent in the interstitium of the normal, healthy kidneys. CD68 is a type I transmembrane glycoprotein and is a known pan-macrophage marker. However, unlike the alveolar macrophages or liver Kupffer cells, which are positive for CD68^14,15^, there were only a few CD68+ macrophages in the healthy kidney tissues. The lack of CD68+ cells in normal kidneys has already been demonstrated in a human protein atlas^16^. In contrast, spindle-shaped CD163+ macrophages were occasionally observed in the interstitium of healthy kidneys, suggesting that these cells represent the resident macrophage population of the kidneys. CD163 is the high-affinity scavenger receptor of the hemoglobin–haptoglobin complex, and is a known marker of the monocyte/macrophage lineage^17^. The phenotype of CD163+ cells is known to resemble alternatively activated, anti-inflammatory M2 macrophages, and several clinicopathologic studies of cancer have demonstrated that CD163+ macrophages are associated with tumor growth/progression and a worse prognosis^18–20^. These data suggest that M2-like resident macrophages perform anti-inflammatory and patrolling functions in healthy kidneys.

In contrast to the healthy control kidneys, the densities of both the CD68+ and the CD163+ macrophages increased significantly during ATN, regardless of their etiology. The location of both subtypes did not overlap in the same tissue section, showing that these two represent distinct subsets of macrophages in human ATN. Because CD68+ cells are hardly seen in healthy kidneys, these cells are likely to be monocytes/macrophages that had migrated from the circulation after injury.

Interestingly, the density of the CD68+ cells in stage 2 or 3 AKI was significantly higher than that in stage 1 AKI, suggesting that this subtype contributes to kidney injury. Given that depletion of monocyte/macrophages using liposome clodronate before IRI resulted in a decrease in the number of kidney macrophages, less severe injury, and inflammation in animal models, the newly appearing CD68+ macrophages in ATN seem to be proinflammatory monocytes/macrophages that participate in injury^10^. However, there was no association between the density of CD163+ macrophages and AKI severity, even though they also increased significantly.

The heterogeneity and plasticity of macrophages is important in injury, repair, and fibrosis in animal models of AKI. This prompted us to investigate the association between different subtypes of macrophages and functional recovery. We found that the density of CD163+ macrophages—but not that of CD68+ cells—was associated with a lack of renal recovery. Older age and an increased density of CD163+ macrophages were associated with a lower 3-month eGFR. Although a direct causal relationship between CD163+ macrophages and progressive fibrosis or a transition to chronic kidney disease (CKD) is not clear from this study, the important role of M2 macrophages in the transition from AKI to CKD has been demonstrated in several animal models. Kim *et al*. (2015) demonstrated that depletion of the predominant M2 macrophages during the recovery phase attenuated interstitial fibrosis, and the adoptive transfer of M2c macrophages after depletion worsened fibrosis^12^. Another study, by Wang *et al*. (2017), demonstrated that M2 macrophages directly transdifferentiated into myofibroblasts and aggravated fibrosis in a chronic allograft nephropathy model^21^. Kaku *et al*. (2014) recently reported that the overexpression of M2 markers, including CD163, CD204, and CD206, on alveolar macrophages occurred in more advanced stages of chronic obstructive pulmonary disease^14^.

In summary, our data suggest that an increase in M2 macrophages might play an important role in the transition from AKI to CKD in human ATN. Given that the biopsies were typically performed relatively late during the recovery phase (3 days after the maximum AKI stage), the persistence of M2 macrophages might lead to progressive fibrosis, as in the animal studies. In contrast, initial severity—whether indicated by the AKI stage based on functional deterioration or by the tubular injury score—was not associated with recovery.

Our study has several inherent limitations. First, unlike in the animal studies, the different timings of the biopsies from the time of the insults makes it difficult to confirm a causal role of macrophages in human AKI. Furthermore, in the deceased donors, it is possible that the transplant microenvironment—including alloimmune stimulation or immunosuppressive drugs—might have affected recovery from ATN, despite the exclusion of patients who subsequently experienced rejection.

However, despite these limitations, we have demonstrated for the first time that different subsets of macrophages might be critically involved in initial injury and the transition from AKI to CKD in human AKI.

## Methods

### Patients

We retrospectively reviewed the medical records of 72 patients who had biopsy-proven ATN but did not have chronic lesions; the biopsies had been conducted between February 2007 and November 2017 on 29 native kidneys and 43 kidneys from deceased donors. All transplants in this study were carried out under the supervision of the Korean Network for Organ Sharing (KONOS) and did not procure organ from prisoners. Donor kidney samples with ATN were obtained by pre-implant wedge biopsy and retrospectively analyzed. The study was approved by the Institutional Review Board of the Korea University College of Medicine (IRB No. 2018AN0189) and was conducted according to the Declaration of Helsinki guidelines. IRB waived the need to obtain informed consent, because the study was retrospective.

### Data collection

We assessed the following data variables: age; sex; diabetes; hypertension; baseline and peak creatinine levels; AKI stage; dialysis; mortality; eGFRs obtained at 1 month and 3 months; and AKI recovery.

The AKI stage was defined according to the Kidney Disease Improving Global Outcomes (KDIGO) criteria. The eGFRs were calculated using the Chronic Kidney Disease Epidemiology Collaboration (CKD-EPI) equation. We defined AKI recovery as a recovery to within 25% of the baseline eGFR during follow-up. Considering the single-kidney function after transplantation, we calculated baseline eGFR of recipient as 70% of donor baseline eGFR.

A renal pathologist—who was unaware of the patients’ clinical information—reviewed the slides that had been stained with hematoxylin and eosin, a periodic acid–Schiff, or Masson’s trichrome stain, to evaluate the following histological factors: glomerulitis, interstitial inflammation, acute tubular necrosis, arteritis, tubular atrophy, and interstitial fibrosis. The factors described above were evaluated in the acquired specimens and scored from 0 to 3.

To calculate the renal macrophage densities, the kidneys were immunostained with anti-CD68 (catalog # ab955; Abcam, Cambridge, MA, USA) and anti-CD163 (catalog # MRQ26; Cell Marque, Rocklin, CA, USA). The immunostained areas were quantified with an interactively set threshold using Fiji software for Windows (National Institutes of Health and the Laboratory for Optical and Computational Instrumentation, USA). Briefly, a color deconvolution was performed to separate the brown color stained area, representing the existence of CD68 or CD163, from a hematoxylin background. The % area exceeding a set threshold, which is considered a positive area, was calculated, and the % areas were averaged in at least three high-power field (x100) images (Supplementary Fig. S2).

### Statistical analysis

The results are presented as the mean ± standard deviation. We compared the numerical data between the groups using the independent sample two tailed *t*-test or one-way analysis of variance (ANOVA). Categorical data were evaluated using the chi-square test or Fisher’s exact test, as appropriate. We used multiple logistic regression models to analyze prognostic factors for outcomes. All statistical analysis was performed using SPSS 20.0 for Windows (IBM Corporation, USA).

## Supplementary information

supplementary files.
