# Supplementary material for: M2 macrophages predict worse long-term outcomes in human acute tubular necrosis
Source: Sci Rep. 2020 Feb 7;10:2122. doi: 10.1038/s41598-020-58725-w (PMC7005727; doi:10.1038/s41598-020-58725-w)
Supplement: Supplementary file 1 — supplementary files. [file 41598_2020_58725_MOESM1_ESM.pdf]

M2 macrophages predict worse long-term outcome in human acute tubular necrosis

Myung-Gyu Kim[1†], Kijoon Lim[1†], Yoo Jin Lee[2], Jihyun Yang[1], Se Won Oh[1], Won Yong Cho[1] and Sang-Kyung Jo[1*]

[1]Division of Nephrology, Department of Internal Medicine, Korea University Anam Hospital, Republic of Korea

[2]Department of Pathology, Korea University Anam Hospital, Republic of Korea

[†]These authors contributed equally to this work.

*Corresponding author:

Sang-Kyung Jo, MD. PhD,

Professor

Department of Internal Medicine, Korea University Medical College

Address: Korea University Anam Hospital, 5Ka, Anam-Dong, Sungbuk-Gu, Seoul, Republic of Korea

Tel: 82-2-920-5475; Fax: 82-2-927-5344; E-mail: sang-kyung@korea.ac.kr

Supplementary Figure S1

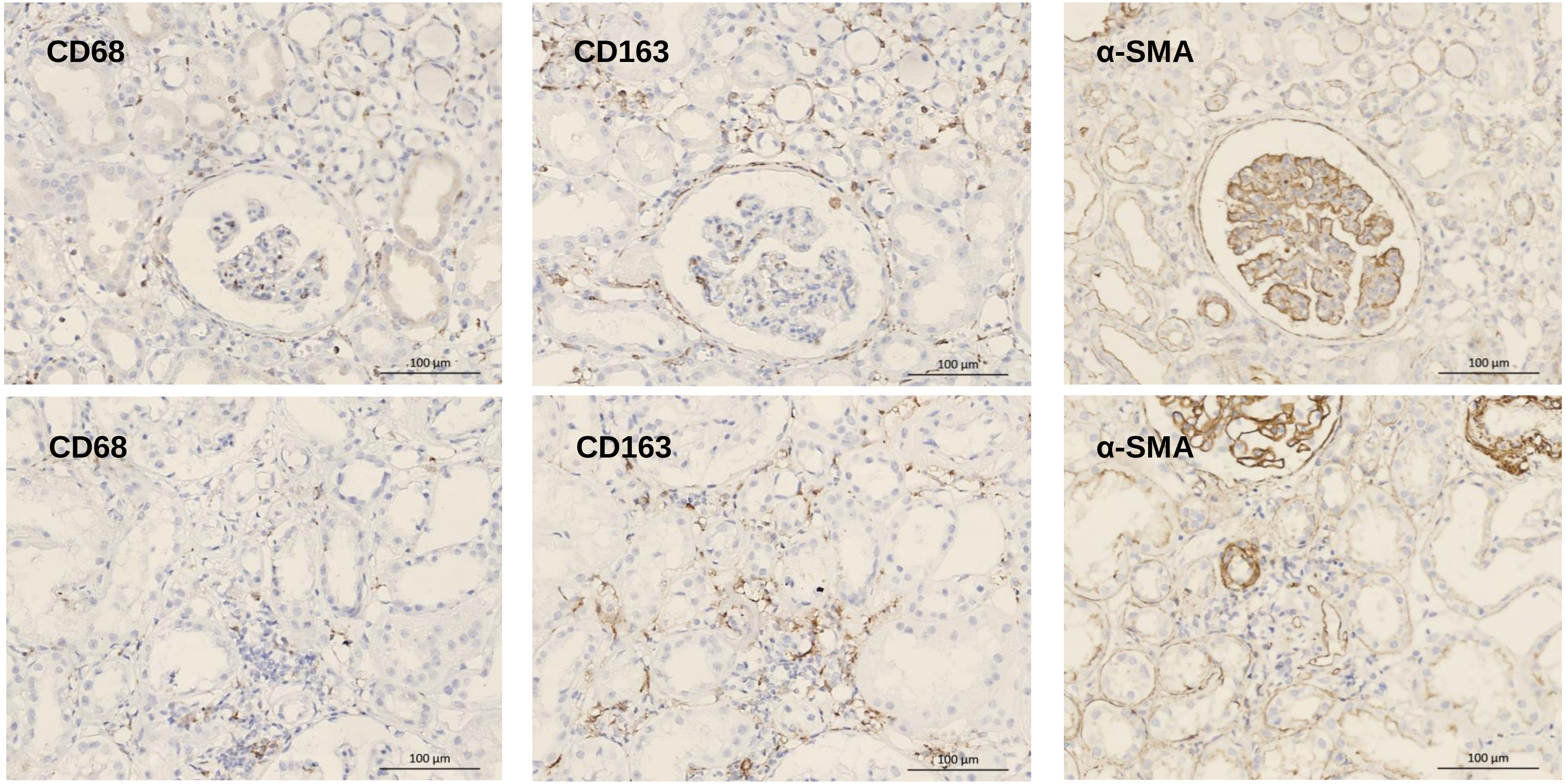

Supplementary Figure S1. Immunohistochemical stainings for CD68, CD163 and α-SMA, α-SMA immunoreactivity was mostly found in glomeruli, arteriole and also in some tubular basement membrane but not in the interstitium where CD68+ and CD163+ cells were found (bar = 100 µm).

Supplementary Figure S2

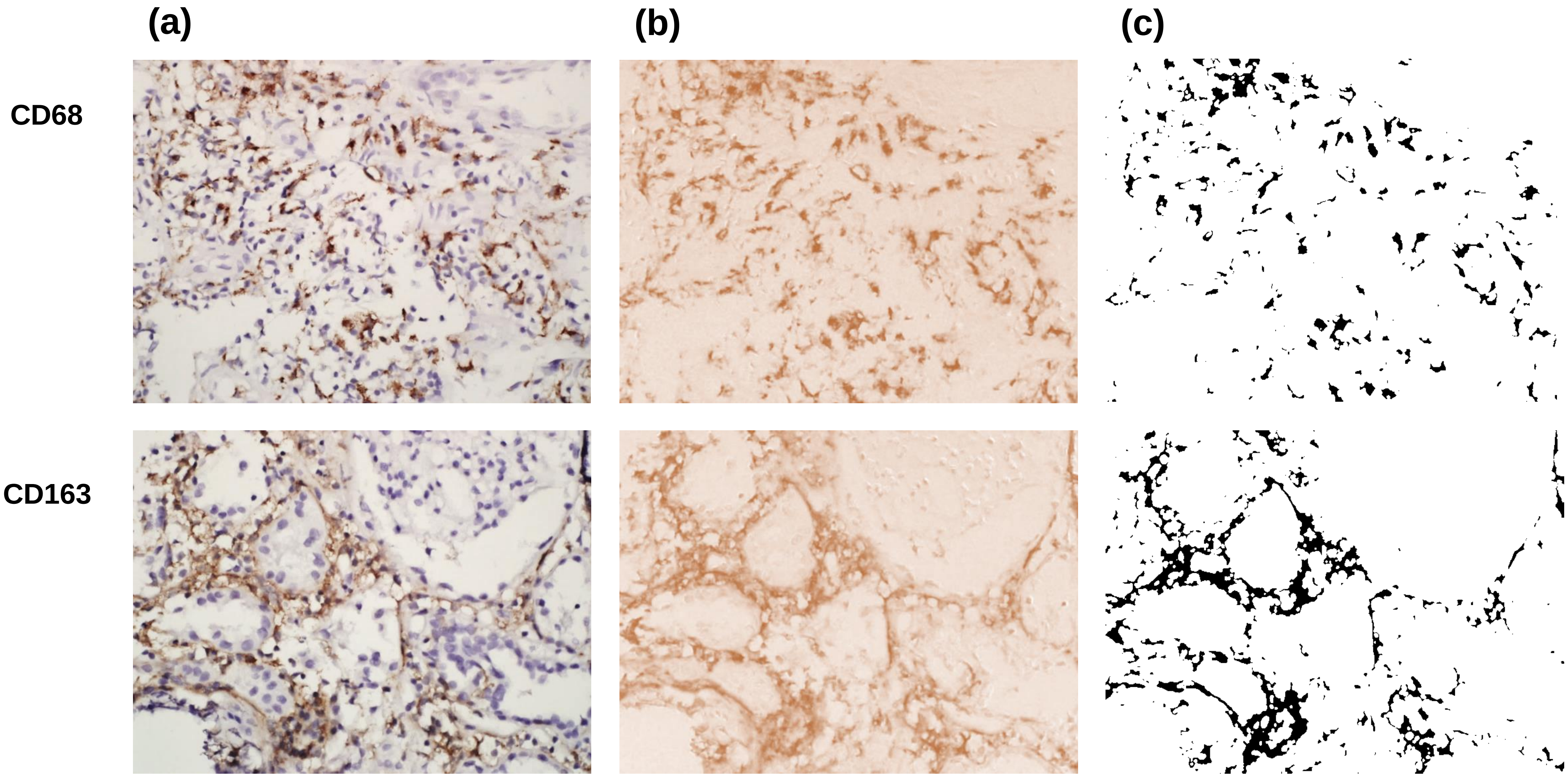

Supplementary Figure S2. Quantification of renal macrophage densities, Using Fiji software, a color deconvolution was performed to separate the brown color stained area, representing the existence of CD68 or CD163, from a hematoxylin background (a, b) and then the area exceeding the set threshold (black color) was quantified (c).

Supplementary Table 1. Impact of CD68+cells infiltration on renal outcome

CD68		1mo eGFR			3mo eGFR			AKI recovery*		
		eGFR <45	eGFR >45	P	eGFR <45	eGFR >45	P	Non-recovery	recovery	P
Total		1.95 ± 2.36	1.42 ± 2.29	0.368	1.19 ± 1.48	1.56 ± 2.13	0.476	1.72 ± 2.17	1.58 ± 2.34	0.831
AKI type	Native AKI	3.21 ± 2.84	2.24 ± 3.15	0.440	2.26 ± 1.46	2.61 ± 2.80	0.815	3.85 ± 2.47	2.28 ± 3.05	0.293
	Deceased donor AKI	1.14 ± 1.63	0.88 ± 1.28	0.573	4.55 ± 2.32	3.47 ± 1.96	0.969	0.54 ± 0.41	1.07 ± 1.53	0.310
AKI severity	Stage 1	0.77 ± 0.60	1.06 ± 1.55	0.635	0.81 ± 0.61	1.09 ± 1.67	0.619	0.57 ± 0.48	1.07 ± 1.47	0.469
	Stage 2,3	2.47 ± 2.66	1.65 ± 2.66	0.332	1.51 ± 1.90	1.89 ± 2.38	0.638	2.36 ± 2.50	1.85 ± 2.68	0.614
biopsy time from peak creatinine	≤2days	1.20 ± 1.84	0.86 ± 1.31	0.514	0.96 ± 1.60	1.02 ± 1.49	0.910	0.44 ± 0.41	1.06 ± 1.58	0.316
	>2days	2.63 ± 2.64	2.20 ± 3.07	0.688	1.60 ± 1.21	2.26 ± 2.64	0.509	2.99 ± 2.49	2.20 ± 2.93	0.517

Values are presented as mean ± standard deviation

*AKI recovery was defined as recovery to within 25% of baseline eGFR during follow-up

Supplementary Table 2. Impact of CD163+ cell infiltration on renal outcome

CD163		1-month eGFR			3-month eGFR			AKI recovery*		
		eGFR < 45	eGFR > 45	P-value	eGFR < 45	eGFR > 45	P-value	Non-recovery	Recovery	P-value
Total		4.89 ± 2.85	3.22 ± 2.10	0.007	4.43 ± 2.40	3.52 ± 1.98	0.116	5.61 ± 3.24	3.44 ± 2.27	0.004
AKI type	Native AKI	5.16 ± 3.64	2.75 ± 2.24	0.039	3.91 ± 3.08	3.61 ± 2.09	0.820	7.54 ± 3.98	2.99 ± 2.44	0.002
	Deceased donor AKI	4.72 ± 2.35	3.53 ± 1.99	0.092	4.55 ± 2.32	3.47 ± 1.96	0.107	4.53 ± 2.32	3.75 ± 2.11	0.343
AKI severity	Stage 1	4.55 ± 2.16	3.40 ± 1.97	0.210	3.77 ± 1.58	3.67 ± 2.34	0.893	4.39 ± 2.09	3.54 ± 2.05	0.418
	Stage 2,3	5.04 ± 3.16	3.11 ± 2.21	0.021	4.98 ± 2.87	3.42 ± 1.73	0.104	6.28 ± 3.66	3.38 ± 2.40	0.006
biopsy time from peak creatinine	≤2days	5.12 ± 2.51	2.96 ± 1.84	0.005	4.54 ± 2.32	3.32 ± 2.01	0.104	4.14 ± 2.01	3.44 ± 2.30	0.458
	>2days	4.68 ± 3.23	3.60 ± 2.42	0.288	4.24 ± 2.69	3.78 ± 1.97	0.631	7.06 ± 3.69	3.44 ± 2.26	0.003

Values are presented as mean ± standard deviation

*AKI recovery was defined as recovery to within 25% of baseline eGFR during follow-up
